# Effect of Highland Barley on Rheological Properties, Textural Properties and Starch Digestibility of Chinese Steamed Bread

**DOI:** 10.3390/foods11081091

**Published:** 2022-04-10

**Authors:** Daying Wu, Liwei Yu, Lei Guo, Shiquan Li, Xiaohua Yao, Youhua Yao, Xinyou Cao, Kunlun Wu, Xin Gao

**Affiliations:** 1 State Key Laboratory of Crop Stress Biology in Arid Areas and College of Agronomy, Northwest A&F University, Yangling 712100, China; wudaying@nwsuaf.edu.cn (D.W.); realylw@163.com (L.Y.); leiguo@nwsuaf.edu.cn (L.G.); lishiquan@nwsuaf.edu.cn (S.L.); 2 State Key Laboratory of Plateau Ecology & Agronomy, Qinghai Key Laboratory of Hulless Barley Genetics and Breeding, Qinghai Subcenter of National Hulless Barley Improvement, Qinghai University, Xining 810016, China; yaoxiaohua009@126.com (X.Y.); youhua8888@126.com (Y.Y.); 3 Crop Research Institute, Shandong Academy of Agricultural Sciences/National Engineering Research Center for Wheat & Maize/Key Laboratory of Wheat Biology and Genetic Improvement in North Yellow & Huai River Valley, Ministry of Agriculture/Shandong Provincial Technology Innovation Center for Wheat, Jinan 250100, China; caoxinyou@126.com

**Keywords:** highland barley, Chinese steamed bread, rheology, mixing, sensory, in vitro digestibility

## Abstract

Highland barley has a different composition and structure to other crops. It has higher contents of total polyphenol (TPC), total flavonoid (TFC) and β-glucan, which can be supplemented to improve the nutrition of wheat-flour-based food. In this study, the flours of three different grain-colored highland barley varieties Beiqing 6 (BQ), Dulihuang (DLH), and Heilaoya (HLY), were added to Jimai60 (JM, a wheat variety with medium gluten) wheat flour at different substitution levels to investigate their effects on the unextractable polymeric protein (UPP) content, micro-structure, rheological properties and mixing properties of dough, and the color, texture, flavor, and in vitro digestion of Chinese steam bread (CSB). The results showed that the moderate substitution of highland barley (20%) increased the UPP%, optimized the micro-structure of gluten, and improved its rheological properties by increasing dough viscoelasticity. The CSBs made from the composite flours exhibited a similar specific volume, cohesiveness, springiness and resilience to wheat CSB, while the firmness of composite CSBs (particularly JM-HLY-20) was delayed during storage. Importantly, the addition of highland barley increased the contents of TPC, TFC and β-glucan, but decreased the in vitro starch digestibility of CSBs. A sensory evaluation showed that JM-HLY CSB was the most preferable. Taken together, highland barley can be used as a fine supplement to food products, with health-promoting properties.

## 1. Introduction

Chinese steamed bread (CSB) is a traditional staple food, with a history of nearly 2000 years in China, and it is increasingly popular among consumers worldwide [1]. CSB is usually made from wheat flour with a medium protein/gluten content and medium gluten strength, which is fermented by yeast [2,3]. Nowadays, food is expected to fulfill more functions and purposes than nutrition and taste. Since the traditional CSB has a high glycemic index (GI), which results in rapid increases in blood glucose concentrations after consumption, people show a strong interest in healthy foods, which can be fortified with promising supplements and ingredients [4]. Therefore, it is of great significance to study CSB fortification and supplementation, to improve its nutritional and functional quality.

Highland barley or hulless barley, as a staple food, is mainly cultivated in Qinghai and Tibetan regions [5]. It is the fourth most-cultivated cereal crop in terms of area in China [6]. Highland barley belongs to the *Triticeae Dumort*, whose grain is similar in shape and structure to wheat grain, but their flours are quite different in their main active components, particularly in terms of phenolics and β-glucan [7]. Phenolic compounds exist in free and bound forms in highland barley, which affect the flavor and appearance of food, and endow food with strong biological activities, such as anti-inflammatory, anti-cancer, hypoglycemic and free radical scavenging activities [8,9,10,11]. Moreover, β-glucan is considered to be the most important bioactive substance in highland barley [12]. As a dietary fiber, β-glucan plays an important role in antibacterial activity, reducing cholesterol level, and relieving constipation [12,13,14]. Due to its biologically active compounds, highland barley has received great attention from researchers as an ingredient of functional food [15]. However, to date, highland barley has not been widely used as a raw material in food processing and even less used in extra-processed diets. Therefore, characterization of the rheological properties of highland barley-wheat dough and physical and nutritional properties of highland barley CSB will shed light on the development of the highland barley industry.

In order to meet the increasing demand for healthy and functional food, researchers have attempted to test new food formulations. For example, a certain proportion of natural plant ingredients were added to common wheat flour to fortify baked foods with biological functions [16,17]. The substitution of red bean powder increases the content of protein and essential amino acids, particularly lysine and threonine, in common CSB [17]. The addition of sorghum flour significantly improves the antioxidant activity of CSB [18], and potato pulp enriches the volatile compounds in CSB [19]. In recent years, highland barley lacking gluten protein has also been supplemented to wheat flour to modify the processing quality [20]. Highland barley flour has been found to improve the network structure of gluten and the sensory quality of noodles [21], and fortify bread and reduce starch hydrolysis and digestibility [22]. The steamed bread supplemented with barley hull and flaxseed hull extract increased the total phenolic content, antioxidant activity and scavenging activity of 2,2-Diphenyl-1-picrylhydrazyl (DPPH) free radicals [23]. However, substituting an inappropriate amount of highland barley flour for wheat flour will negatively affect the quality of wheat flour [24]. Therefore, it is particularly important to determine the appropriate replacement ratio of highland barley to improve the processing, nutritional and baking quality of wheat flour.

Most of the raw materials were basically commercial flours in previous studies, which investigate the effect of highland barley on the physiochemical and rheological properties of wheat dough [21,22,23,24]. However, how the added highland barley varieties affect the physiochemical and rheological properties of wheat dough is not clear and needs further investigation. In this study, flours of three different-colored highland barley varieties were selected and individually added to wheat flour (a medium gluten wheat variety Jimai60) with different ratios, and the proportion of unextractable polymeric protein (UPP%), micro-structure, rheological properties and mixing properties of dough were determined. The color, texture, flavor and in vitro digestion of CSBs made from Jimai60 formulated with the three highland barley flours was investigated. This study will provide helpful information for processing barley-supplemented food products, taking different highland barley varieties into consideration.

## 2. Materials and Methods

### 2.1. Materials

Jimai60 (JM), a wheat variety with medium gluten, and three highland barley varieties Beiqing 6 (BQ, a white-grained variety), Dulihuang (DLH, a yellow-grained variety), and Heilaoya (HLY, a black-grained variety) were planted and harvested at the experimental station of Northwest A&F University (Yangling, Shaanxi, 108°4′ E, 34°16′ N) in 2019–2020 growing season. After being sun-dried, the grains were tempered and ground into flour with a Brabender Quadrumat Senior (Brabender Instruments, Hackensack, NJ, USA), and then sieved (100-mesh). Flours of the three highland barley varieties were individually added to wheat flour at the substitution levels of 0:100, 10:90, 20:80, 30:70, and 100:0 for reconstitution, and the composite flours were placed in a refrigerator at 4 °C for further analyses.

### 2.2. Determination of the Grain Quality of the Wheat and Three Highland Barley Varieties

#### 2.2.1. Determination of the Basic Components in Grains of Wheat and the Three Highland Barley Varieties

##### The Main Chemical Compositions

The near-infrared reflectance (NIR) spectrometer (Diode Array 7250 Perten, Huddinge, Sweden) was used to determine the moisture, protein and starch contents in grains of wheat and the three highland barley varieties. Each sample was tested three times.

##### Analysis of Size Distribution of B-Type Starch Granules

The starches of the wheat and three highland barley varieties were extracted according to the method of Liu et al. [25], with some modifications. The flour (10 g) was poured into distilled water (6 mL) and fully kneaded, and the dough was placed under running water and kneaded until no starch was washed out. The starch slurry was filtered twice with eight layers of 20-mesh gauze, and the filtrate was centrifuged (4000× *g*, 10 min) after standing for 8 h. The precipitate was washed three times with 75% ethanol, and then dried in an oven (40 °C, 36 h).

The dried starch was ground in a mortar and pestle and then passed through a 200-mesh sieve to obtain starch granules with a diameter of less than 0.075 mm.

The particle size distribution of starch extracted from the wheat and highland barley flours was measured with a laser diffraction analyzer (Microtrac S3500 SI, Microtrac Inc., Largo, FL, USA), and the percentage of starch with a particle size ≤10 μm was calculated as the proportion of B-type starch granules in total starch [26]. Each sample was tested thrice.

##### Amylose Content

The amylose content in the wheat and highland barley flours was determined by the Amylose/Amylopectin Assay Kit (Megazyme International Ireland Ltd., Bray, Ireland) with three replicates for each sample [22].

##### The Ratio of High-Molecular-Weight to Low-Molecular-Weight Glutenin Subunits (H/L)

The ratio of high-molecular-weight (HMW-GS) to low-molecular-weight glutenin subunits (LMW-GS) (H/L) in the wheat and highland barley flours was determined by reverse-phase, high-performance liquid chromatography (RP-HPLC) according to the method previously reported by Li et al. [27]. The chromatographic column used was ZORBAX SB-C18 (Agilent, Palo Alto, CA, USA), and the mobile phases A and B of the elution system were deionized water and acetonitrile solutions, with each containing 0.08% trifluoroacetic acid. Each sample was measured in triplicate. The formula for calculating H/L is as follows:

$$H/L=\frac{\mathrm{HMW}-\mathrm{GSs}\;\mathrm{area}}{\mathrm{LMW}-\mathrm{GSs}\;\mathrm{area}}\;\times \;100\%$$

#### 2.2.2. Determination of the Nutritional Ingredients of the Wheat and Three Highland Barley Varieties

##### β-Glucan Content

According to the method provided by the Megazyme kit (Megazyme International Ireland Ltd., Bray, Ireland), β-glucan content in the wheat and highland barley flours was determined thrice by mixed-linkage analysis.

##### Total Polyphenol Content (TPC)

TPC of the wheat and highland barley flours was determined using the colorimetric method with minor modifications, namely, the Folin–Ciocalteu reagent method, according to the process described by Shen et al. [11]. Each flour (1.5 g) was mixed with 15 mL methanol solution (70%), and centrifuged at 6000× *g* for 15 min after 2 h. The supernatant (0.2 mL) was added to 1 mL Folin phenol reagent. After 5 min, 3 mL sodium carbonate solution was added to the mixture. After being kept in the dark at room temperature for 15 min, the optical density of the mixture was measured at 725 nm using an ultraviolet spectrophotometer (Shimadzu UV-1800, Kyoto, Japan), with gallic acid as the standard curve. The TPC of the samples was described by gallic acid equivalent (mg GAE/g).

##### Total Flavonoid Content (TFC)

TFC of the wheat and highland barley flours was determined according to the previously reported method with minor modifications [28]. Flavonoid in the flour samples was extracted with 80% methanol solution at 25 °C for 4 h, followed by centrifugation at 6000× *g* for 15 min. An equal amount (1 mL) of each extract or standard solution of rutin was mixed with 0.15 mL NaNO_2_ solution (50 mg/mL), and 0.15 mL Al(NO_3_)_3_ solution (100 mg/mL) was added. After 6 min, the mixture was added with 2 mL NaOH solution (40 mg/mL) and incubated at room temperature for 15 min. The absorbance of the rutin standard solution and the mixture was measured by an ultraviolet spectrophotometer (Shimadzu UV-1800, Kyoto, Japan) at 517 nm. TFC in the samples was estimated as rutin equivalent (mg GAE/g).

### 2.3. Determination of Quality Characteristics of Composite Powder

#### 2.3.1. UPP% and the Ratio of Glutenin to Gliadin (Glu/Gli)

The preparation of SDS extractable and unextractable proteins (EPP and UPP) was conducted according to the method established by Singh and Singh [29]. The flour sample (0.025 g) mixed with SDS-Na phosphate buffer (1 mL, pH = 6.9) was placed in a water bath at 30 °C and then centrifuged (13,000× *g*, 10 min) to obtain EPP. The precipitate was ultrasonically extracted in phosphate buffer (1 mL, pH = 6.9) for 30 s and centrifuged (13,000× *g*, 10 min) to obtain UPP. The extracted EPP and UPP were filtered through a 0.45 μm filter before detection.

Both EPP and UPP extracts were illustrated by size-exclusion, high-performance liquid chromatography (SE-HPLC), according to the method reported by Liu et al. [25]. The calculation formulations for UPP% and Glu/Gli were as follows:

$$\mathrm{UPP}\%=\frac{\mathrm{UPP}\;\mathrm{area}}{(\mathrm{EPP}+\mathrm{UPP})\;\mathrm{area}}\times 100\%$$

$$\mathrm{Glu}/\mathrm{Gli}=\frac{\mathrm{Glutenins}\;\mathrm{area}}{\mathrm{Gliadins}\;\mathrm{area}}\times 100\%$$

#### 2.3.2. Micro-Structure of Gluten

The micro-structure of the gluten in doughs was observed by confocal laser scanning microscopy (CLSM), as reported by Gao et al. [30]. The rhodamine B solution (1.2 mL, 0.01 g/mL) was added to flour sample (2 g) and, after being thoroughly kneaded, the dough was rested at 25 °C for 10 min to allow the dough to be evenly dyed. The dough sample was observed by a CLSM (IX83-FV1200, Olympus, Tokyo, Japan) with a semiconductor laser LD559. Five images of gluten micro-structure were randomly captured for each sample, with a resolution of 512 × 512 pixels and a size of 211.5 × 211.5 μm. The images were processed with AngioTool64 (version 0.6a, National Cancer Institute, Bethesda, MD, USA) according to four parameters: protein area, total protein length, protein end points, and lacunarity.

#### 2.3.3. Rheological Properties of Dough

The rheological properties of dough were measured using a rheometer (DHR-1, TA Instruments, New Castle, DE, USA) equipped with a 40-mm parallel metal plate probe. The dough was placed in the center of the round platform of the rheometer and, after the gap was adjusted to 1 mm, silicone oil was applied to the edge of the dough sample to prevent moisture from evaporating during the test. Under the conditions of 25 °C and a frequency range of 0.1–10 Hz, the storage modulus (G′), and loss modulus (G″) were measured by frequency sweep tests at a strain of 0.1%, and the loss tangent (tan δ) was calculated as the ratio of G″ to G′ [31]. Each sample was measured in triplicate.

#### 2.3.4. Mixing Properties of Dough

The mixing properties of dough were measured by Mixolab2 (Chopin Technologies, Tripette and Renaud, Paris, France). The procedure was set referring to the method reported by Zhang, Mu, and Sun [32]. The measurement was made over three stages: constant temperature, heating and cooling stage. Nine parameters for protein and starch properties were determined during different periods [33]: water absorption (%), dough development time (min), dough stability time (min), maximum dough consistency (C1, Nm), protein weakening (C2, Nm), starch gelatinization maximum viscosity (C3, Nm), starch gelatinization thermal stability (C4, Nm), starch retrogradation characteristics (C5, Nm), and gelatinization temperature (°C). The analysis was repeated thrice for each flour sample.

### 2.4. Determination of the Composite CSBs’ Characteristics

#### 2.4.1. Preparation of the Composite CSBs

For highland barley-wheat flour (50 g), yeast (0.5 g) and a moderate amount of water (at 30 °C, the amount of water added was based on the water absorption in the mixing properties test) were used as raw materials for making CSB. All the ingredients were stirred in the mixing bowl for 10 min to form a homogeneous dough and fermented in a fermentation tank (30 °C with relative humidity from 80% to 85%) for 1 h. The fermented dough was kneaded into a smooth hemisphere and steamed in a steamer over boiling water for 20 min. The CSB was cooled to room temperature for further tests.

#### 2.4.2. Specific Volume Determination

The highland barley-wheat CSB was weighed, and the volume of the steamed bread was measured by the millet replacement method [34]. The diameter of the bottom side of the steamed bread and its height were measured. The specific volume of CSB was calculated as the ratio of volume to weight. Each sample was analyzed in three replicates.

#### 2.4.3. Textural Profile Analysis

The textural properties of highland barley-wheat CSB were analyzed using Texture Analyzer (TVT6700, Perten, Huddinge, Sweden) equipped with a 25-mm-diameter probe P-CY25S referring to the method of Guo, Yang, and Zhu [34] with minor modifications. The CSB was sliced vertically from the middle to obtain a 20-mm-thick uniform slice with a larger diameter than that of the probe, which was placed in the center of the platform, and then compressed twice to 80% of the original thickness with a 3 s hold-period between compressions, by a trigger compression force of 5 g with the pre-test speed, post-test speed and test speed set at 1 mm/s, 1 mm/s and 1.7 mm/s. The average values of firmness, cohesiveness, springiness and resilience were obtained through three replicates.

#### 2.4.4. In Vitro Starch Digestibility

The in vitro starch digestibility of the CSB samples was determined according to the method established by Toutounji et al. [35]. The freeze-dried CSB sample was ground into powder (200 mg) and suspended in 15 mL sodium acetate buffer (0.2 M, pH 5.2), and mixed with five glass beads (5 mm) and 15 mL working enzyme solution (120 U/mL α-amylase and 80 U/mL amyloglucosidase in sodium acetate buffer). The mixture was shaken in a shaker (200 rpm) at 37 °C to hydrolyze. Digestion solution (0.2 mL) was collected at 0, 30, 60, 90, 120, and 180 min, and immediately heated in boiling water for 10 min. The glucose content in the supernatant, after centrifugation for 10 min, was determined using D-glucose analysis kit (Megazyme International Ireland Ltd., Bray, Ireland). Each sample was assayed in triplicate at each timepoint.

#### 2.4.5. Sensory Evaluation

The scoring system for sensory quality of CSBs was used, referring to the Chinese Standard GB/T 35991-2018 (Table S1). The discriminative testing was conducted in triplicate by five trained participants, who took CSBs as part of their diet. Each participant evaluated the specific volume (20 points), surface structure (10 points), color (10 points), appearance shape (10 points), internal structure (15 points), elasticity (15 points), viscidity (10 points) and flavor (10 points) of four CSBs samples, and the total score (100 points) was used to judge the overall sensory quality of CSBs. The participants were required to gargle with water between the two samples.

### 2.5. Statistical Analysis

All data were described as mean ± standard deviation. Differences in test values between different samples were calculated by SPSS Statistical Software (SPSS Version 21.0, IBM SPSS) for a one-way analysis of variance (ANOVA) and LSD test at a 95% confidence interval.

## 3. Results and Discussion

### 3.1. Grain Quality of the Wheat and Three Highland Barley Varieties

#### 3.1.1. Basic Components in the Grains of the Wheat and Three Highland Barley Varieties

The basic components in the grains of the wheat and three highland barley varieties are shown in Table 1. The three highland barley grains had a slightly lower protein content (14.40%, 9.11%, and 15.27%) than the wheat grain (15.48%). Similarly, the highland barley grains also showed a relatively lower H/L ratio, which was attributed to the lower content of high-molecular-weight glutenin subunit in the three highland barleys [5]. Among the three highland barleys, BQ showed the highest H/L ratio, followed by DLH, with HLY showing the least. In terms of starch properties, BQ showed the highest total starch content (61.55%); while BQ had the lowest size distribution (number proportion) of B-type starch granules and amylose content (42.49% and 28.28%), with no significant difference compared to HLY, and DLH had the highest size distribution (number proportion) of B-type starch granules and amylose content, i.e., 56.93% and 34.92% (Table 1). The variations in protein and starch contents among the three highland barley varieties may have different effects on the processing quality of composite flours. Among all the grains, BQ and JM showed no significant difference in the content of the total starch and size distribution (number proportion) of B-type starch granules; HLY and DLH had a lower total starch content than JM; and all the highland barley varieties showed a higher size distribution (number proportion) of B-type starch granules and slightly higher amylose content than JM. According to a recent study showing that B-type starch promoted the formation of gluten network structures by interacting with gluten protein [36], and amylose content played an important role in the pasting properties of flour [37], the processing quality of CSBs may be improved by adding highland barley flour enriched with B-type starch and amylose.

#### 3.1.2. Nutrients of the Wheat and Three Highland Barley Varieties

The analysis of nutrients of JM and the three highland barley varieties showed that β-glucan content, TPC and TFC of the three highland barley varieties ranged from 3.60% to 4.15%, 1.02 to 1.24 mg GAE·g^−1^, and 1.05 to 1.74 mg GAE·g^−1^, respectively, which were significantly higher than those of JM (Table 1). There were some differences in the content of nutrients among the three highland barley varieties. BQ showed the highest content of β-glucan (4.15%), whereas DLH and HLY had lower β-glucan content, 3.83% and 3.60%, respectively, which may be attributed to the different genotypes [10,38]. Previous studies have shown that β-glucan in highland barley showed a strong digestion resistance [39], and polyphenols and flavonoids are also antioxidants [11]. Therefore, the nutritional quality of the currently studied CSB composite is supposed to be effectively improved.

### 3.2. Quality Characteristics of Composite Powder

#### 3.2.1. The Glutenins to Gliadins Ratio (Glu/Gli) and UPP%

**Figure 1:**
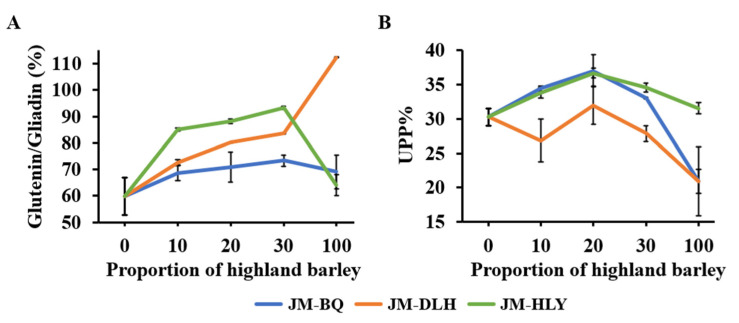
The dynamic variation of the ratio between gliadin and glutenin (**A**) and UPP% (**B**) of composite flour combinations.

In order to determine the effect of the added highland barley flour on the protein aggregation of the wheat flours, Glu/Gli and UPP% of the composite flour combinations were determined by HPLC (Figure 1). The Glu/Gli of the composite flours varied greatly among the highland barley varieties, with JM-HLY flour exhibiting the highest Glu/Gli, whereas JM-BQ flour exhibited the lowest (Figure 1A). At a moderate addition ratio, the Glu/Gli of the composite flours increased when the highland barley substitution level increased, which is attributed to the higher Glu/Gli values of the three highland barleys, compared to that of JM. Glu/Gli is an important factor in physiochemical and rheological properties of wheat flour and dough. It has an appreciable effect on viscoelasticity, stability and development time of dough [40]; it also affects the structural properties and elasticity of wheat food, since a change in Glu/Gli affects the gelatinization, thermal and structural properties of starch [41]. It is, therefore, particularly important to select an appropriate composite combination to improve the processing properties of CSB. UPP% has proved to be a better index than total glutenin to characterize the processing quality of dough [42]. As shown in Figure 1B, the three highland barley flours showed a lower or similar UPP% compared to JM flour. The substitution of highland barleys generally increased the UPP% of the composite flours, except for 10% and 30% JM-DLH combinations. The HLY flour and JM-HLY combinations showed the highest UPP%; while the BQ and DLH flours showed a lower UPP%; JM-BQ combinations show a higher UPP% than JM-DLH combinations. This may be attributed to the higher total protein content in the BQ flour and HLY flour (Table 1).

The polymeric proteins are mainly composed of glutenin linked by disulfide bonds, and the sulfhydryl/disulfide exchange reactions led to the depolymerization and rearrangement of glutenin polymers [43]. Therefore, in this study, the increase in the UPP of composite flours may be due to the rearrangement of glutenin polymers caused by the linking of gliadin and glutenin in wheat and highland barley, thus increasing the size of glutenin polymers. In addition, the change in UPP% affects the rheological properties of flour [29], and has a significantly positive correlation with the strength and elasticity of wheat dough [44,45]. It can be inferred that adding a moderate amount of highland barley flour can increase the dough strength.

#### 3.2.2. Micro-Structure of Gluten

**Figure 2:**
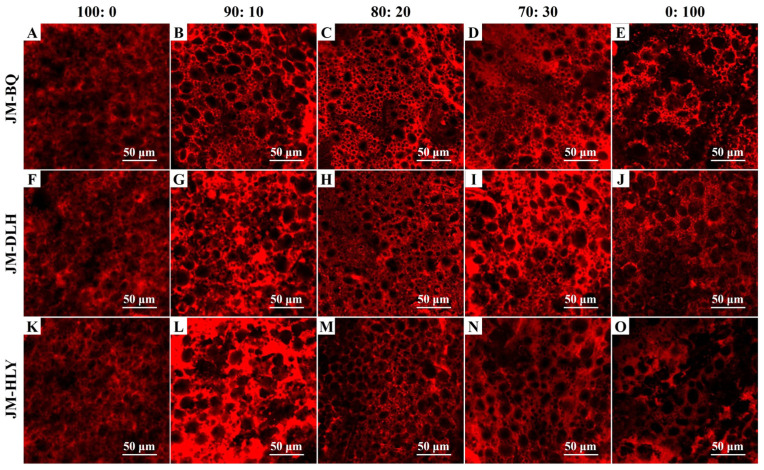
The gluten micro-structure of composite dough samples added with three highland barley varieties with different ratios: (**A**–**E**): JM-BQ; (**F**–**J**): JM-DLH; (**K**–**O**): JM-HLY. The samples stained with Rhodamine B and visualized by confocal laser scanning microscopy. Scale bar = 50 µm.

**Figure 3:**
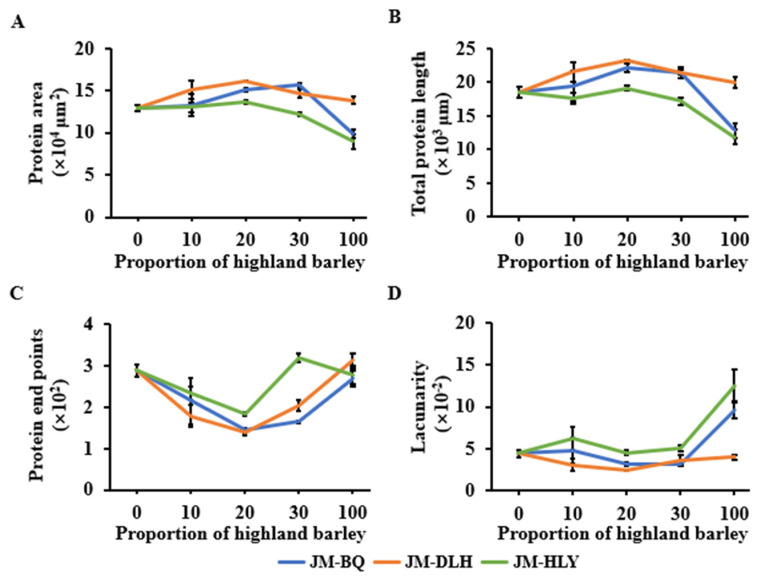
Quantitative analysis of the gluten network in highland barley composite dough samples determined by AngioTool software. (**A**) protein area. (**B**) total protein length. (**C**) protein endpoints. (**D**) lacunarity.

To further determine the effect of added highland barley on the gluten structure of dough, the gluten micro-structure of the composite dough was observed (Figure 2). In the photomicrographs of JM dough (Figure 2A,F,K), a continuous gluten network was observed, while the photomicrographs of highland barley showed a loose and discontinuous structure (Figure 2E,G,O). It is clear that the added highland barley affected the micro-structure of the dough. When the replacement of highland barley ranged from 10% to 20%, the gluten network structure was improved compared with the control; when the replacement level was 20%, the gluten network was well-developed, with the continuous structure highlighted in red in the graphs and fewer openings in the network (Figure 2C,H,M), which may be attributed to more B-type starch granules being introduced by highland barley flours (Table 1) [36]. On the other hand, when the highland barley substitution level reached 30%, the protein network was destroyed and became less continuous (Figure 2D,I,N), which is consistent with the fact that the UPP% of the composite flour decreased in the process. The main reason may be that the high proportion of highland barley reduced the glutenin content in the composite flour, and thus cleaved the disulfide bonds to free sulfhydryl groups, which, in consequence, reduced the gluten protein formed by the disulfide bond in the dough system [46]. Further quantitative analysis showed that the partial replacement of BQ or DLH flour increased the protein area and total protein length of the doughs, while the partial replacement of the HLY flour was less effective (Figure 3A,B). The substitution of highland barleys generally decreased the protein end points and lacunarity of the doughs (Figure 3C,D), especially at the replacement level of 20%, which is in agreement with the results regarding the UPP% of the abovementioned formulations. The DLH and BQ flours improved the micro-structure of the dough more than HLY, which can be explained by the fact that the DLH and BQ showed higher H/L ratio, β-glucan content and Glu/Gli than the HLY. It has been reported that the fibers such as β-glucan in the highland barley interact with gluten through hydrogen bonds, which act as a filler in the network structure, and is responsible for the improved composite dough structure [47,48]. Fewer protein endpoints and a smaller lacunarity indicate the better cohesion and consistency of the gluten network, indicating a denser gluten structure of the dough [49]. Therefore, the improved gluten structure of the composite doughs is supposed to have improved processing qualities.

#### 3.2.3. Rheological Properties

**Figure 4:**
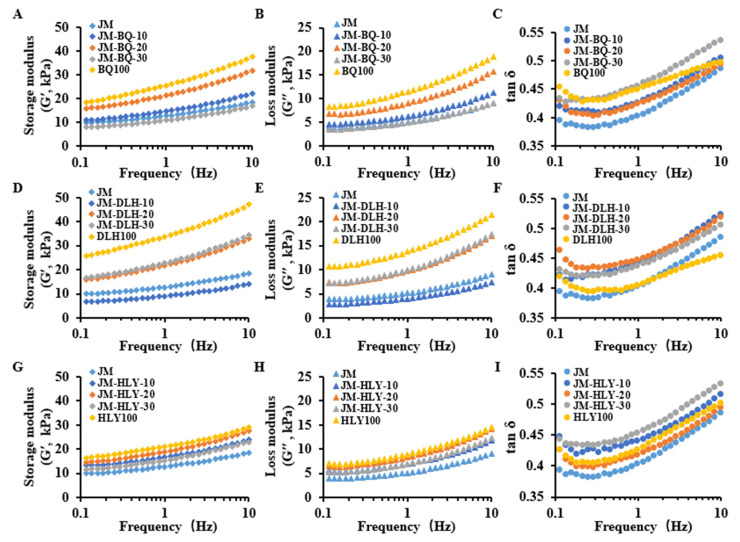
Rheological properties of highland barley composite dough. (**A**,**D**,**G**): storage modulus (G′); (**B**,**E**,**H**): loss modulus (G″); (**C**,**F**,**I**): tan δ (G″/G′).

G′ represents the elastic modulus, and G″ represents the viscous modulus. When G′ > G″, the dough exhibits the dominant elastic behavior; when G′ < G″, it exhibits dominant viscous behavior [50]. The effect of different substitution levels of highland barley flour on the rheological behavior of dough is shown in Figure 4. Except for JM-BQ-30 and JM-DLH-10, the dynamic modulus (G′ and G″) for all composite doughs were relatively higher than those for wheat dough, indicating that a moderate replacement of wheat flour with highland barley flour increased the viscosity and elasticity behavior of the dough samples. Among the results for the three highland barley varieties, JM-DLH dough showed the highest values of G′ and G″, followed by JM-BQ dough, with JM-HLY dough showing the least. These elastic-viscous results are in agreement with the results of Glu/Gli and gluten micro-structure of the three highland barley varieties, indicating that the gluten content of highland barley had a certain positive effect on the rheological properties of dough. Among the barley–wheat combinations, JM-BQ-20 and JM-HLY-20 doughs exhibited significant increases in G’ and G″, while, among JM-DLH composite combinations, JM-DLH-30 dough showed the highest G′ and G″. Both G’ and G″ were in linear relation to frequency, and all dough samples showed greater G′ than G″ within the frequency ranging from 0.1 to 10 Hz; that is, the value of tan δ was less than 1, indicating that the composite dough showed the dominant elastic behavior [31]. This may be attributed to the presence of the gluten network in the dough system, which made the dough elastic and solid [51].

Tan δ, the ratio of G″ to G′, with values ranging from 0.1 to 1, indicates that dough is a weak gel [52]. All the composite doughs showed a higher tan δ than JM dough (Figure 4C,F,I). The current results are in line with the reports of Izydorczyk et al. [53], who found that the addition of barley significantly changed the value of viscoelastic modulus of the dough and caused the tan δ to increase. In addition, with the increase in vibration frequency, the tan δ value of the composite flour decreased and then gradually increased, which means that the viscosity of dough gradually played a role in the higher vibration frequency. The dough may be softened due to the high content of starch in highland barley [54]. However, the doughs of different wheat barley composite combinations and formulations had a different tan δ and did not show an obvious pattern of change when highland barley addition varied. When the substitution of highland barley was 10% or 30%, the tan δ for JM-HLY dough was the highest; while, when the substitution was 20%, the tan δ value was the lowest. As a smaller tan δ value indicates a higher G′ of the dough system, the appearance quality of JM-HLY-20 CSB may be better. Given the fact that the cross-linkage of polymer systems impacts the storage modulus [55], it is speculated that the different rheological performances may be attributed to the various cross-linkage levels of starch and protein among the different composite combinations and formulations. Moreover, B-type starch can improve the tensile resistance and hardness of wheat dough by promoting the continuous formation of a gluten network [36], and the starch granules in the composite dough may be surrounded by β-glucan chains, which also contributes to an increase in G′ and G″, replacing a large number of free water regions [56,57]. Therefore, the variations in rheological behavior in different composite combinations can be explained by more B-type starch and β-glucan in the composite dough because of the substitution of highland barleys. As a result, the substitution of highland barley flours increased the dough viscoelasticity, which thus improved the micro-structure of dough.

#### 3.2.4. Mixing Properties

**Figure 5:**
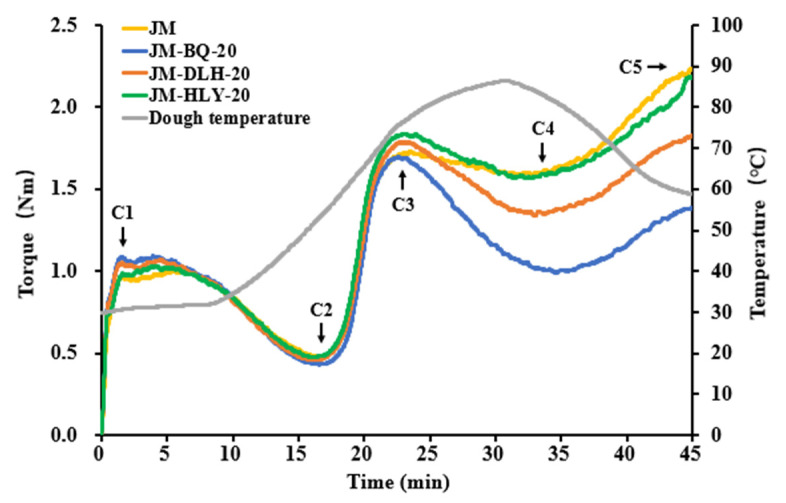
The mixing profiles of highland barley composite dough samples. C1: dough development; C2: protein weakening; C3: starch gelatinization; C4: cooking stability; C5: final viscosity.

The mixing properties of JM-BQ-20, JM-DLH-20 and JM-HLY-20 doughs were measured to further determine the effect of the moderate substitution of barleys on the quality of the final product. As shown in Figure 5, four different Mixolab curves were obtained. No significant trends were observed among the first four samples in 23 min, indicating that highland barley had little effect on the protein characteristics of dough; whereas the curve showed a significant consistency difference after 23 min, indicating that the addition of highland barley flour had an impact on the starch properties of dough (Table 2).

At C1, when the torque value was 1.1 ± 0.05 Nm, the water absorption for the composite doughs increased after the addition of highland barley, which was attributed to the increased hydrophilic groups in dietary fiber and stronger binding with water molecules [58]. Dough development time and stability time, reflecting the strength of the protein network structure in the process of dough mixing [33], were significantly affected by barley addition. The dough development time for JM was 6.08 min, which was longer than that for JM-BQ-20 (4.12 min), JM-DLH-20 (4.73 min) and JM-HLY-20 (4.19 min). Given that the longer the dough is formed, the more energy is put into its production, the composite flours may reduce energy consumption in the food processing industry [59]. Similarly, the stability time for JM dough was 8.45 min, which was slightly longer than that for JM-BQ-20 (7.26 min), JM-DLH-20 (7.53 min) and JM-HLY-20 (7.84 min). There was no significant difference in development time and stability time for the three highland barley composite doughs. A slight decrease in the development time and stability time of the composite doughs showed that the substitution of highland barley at a certain level weakened the gluten strength of all-purpose wheat and reduced the mixing resistance of gluten, but its negative effect on the mixing quality of the dough and physical quality of the CSB was minimal. The reason for this may be the reduction in disulfide bonds in the dough system [46], which agrees with the report by Rosell et al. [60] on the partial replacement of wheat flour with quinoa flour (12.5–25%).

After further mixing with increasing temperature, the protein continued to weaken, and minimum torque (C2) was reached. Among the four samples, JM dough showed the highest torque value at C2, indicating that the quality of protein became worse with the addition of highland barley, but was still within an acceptable range. The process after C2 was starch gelatinization with the change in temperature. At the heating stage, the rapid water absorption and expansion of starch particles in the dough leads to the dissociation of a large amount of amylose, which increases the viscosity of the dough system and increases the torque to its peak (C3) [61]. In addition, the gelatinization viscosity of barley starch is higher than that of wheat [62]. As the process continues, the dough viscosity decreases due to the breakdown of free starch catalyzed by amylase, resulting in a minimum torque (C4), which represents the stability of the hot starch paste. During the cooling stage, the dough viscosity rises again due to the retrogradation of starch, until the final torque (C5) occurs at the end of the mixing process [63]. The process from C4 to C5 represents the retrogradation of starch. It can be seen that the C4 and C5 values of the composite doughs were smaller than those of JM dough, indicating that the addition of highland barley weakened the stability of starch in the dough system on the one hand, and prevented the starch from aging on the other hand. The values of C3, C4 and C5 for JM-HLY-20 dough were the largest, followed by those of JM-DLH-20 dough and JM-BQ-20 dough (Table 2). These results were in inverse proportion to the total starch content, β-glucan content and TPC of the three highland barley doughs reported in Section 3.1.1, whereas they were generally linear in relation to the amylose content and size distribution (number proportion) of B-type starch granules. Previous studies have shown that the addition of a certain amount of barley to wheat flour reduced the retrogradation of starch [20,24], which may be attributed to the higher levels of β-glucan and polyphenols in highland barley [57,64].

### 3.3. The Quality Characteristics of Composite CSBs

#### 3.3.1. Specific Volume and Sensory Analysis

**Figure 6:**
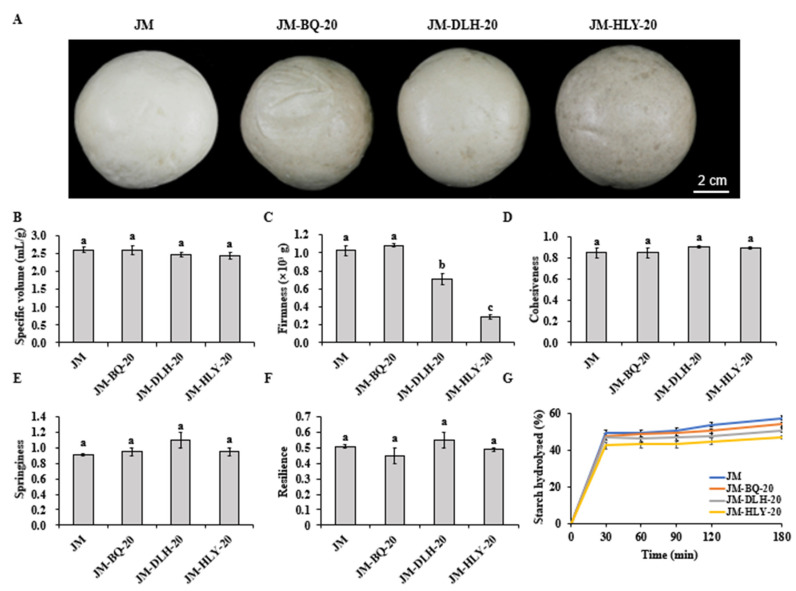
The baking performance (**A**,**B**), textural properties (**C**–**F**) and starch digestibility (**G**) of highland barley composite CSBs. Different letters above the columns for specific volume, firmness, cohesiveness, springiness, and resilience indicate significant differences at *p* < 0.05.

In order to further estimate the effect of highland barley addition on the processing properties of the wheat dough, the flour formulations were used to make CSBs. Figure 6B shows that the substitution levels of BQ, DLH and HLY flours had no significant effect on the specific volume of the CSB. The specific volume reflects the expansion degree of the CSB to a certain extent, which is related to the softness and chewing characteristics of steamed bread [65]. The specific volume of the CSB mainly depends on the formation and expansion of gluten network, which is the main parameter that characterizes the volume expansion and air retention of the dough during the fermentation process [66]. Too large a specific volume means a very open granular structure, while too small specific volume indicates a compact and closed dough structure [67]. The results show that a 20% highland barley substitution had a positive effect on wheat dough gluten, and the substitution of barley flours did not significantly affect the specific volumes of the four CSBs (Figure 2 and Figure 3).

The sensory quality of the CSB samples was also evaluated (Table 3). The composite CSBs obtained lower scores for surface structure (not round in form) and internal structure than the wheat CSB, which is consistent with the previous study showing that the surface of noodles made from composite flours of wheat and hulless barley is rough [21]. In addition, the color of the composite CSBs, particularly JM-HLY-20 formulation, was slightly dark (Figure 6A), which was attributed to the fact that HLY is a black-grained variety, while BQ is a white-grained variety and DLH is a yellow-grained variety. In terms of elasticity, viscidity and flavor, no significant difference was found between the JM-HLY-20 and JM CSBs, while the scores of JM-BQ-20 and JM-DLH-20 CSBs were slightly lower, which may be related to the differences in substitution levels in the highland barley combinations. The final evaluation results showed that the sensory total score of JM-HLY-20 CSB was highest, indicating that it is a preferable formulation.

#### 3.3.2. Textural Properties

Textural properties are important indicators for evaluating food-chewing sensory performance [68]. The TPA results of the four CSB samples showed that a 20% highland barley substitution had a great effect on the hardness of the CSB (Figure 6C). When comparing composite CSB with pure wheat CSB, we found that JM-DLH-20 and JM-HLY-20 CSBs showed a lower firmness than JM CSB, whereas JM-BQ-20 and JM CSBs showed similar firmness. When comparing the three barley composite breads, we found that the JM-HLY-20 CSB showed the lowest firmness, while the JM-BQ-20 CSB showed the highest, which was consistent with the results of the dough-mixing properties. The differences in firmness may be related to the various tan δ values. The increase in firmness indicates the aging of CSB, which is usually caused by the disintegration of starch molecules from gluten network [69]. However, the addition of highland barley had no significant effect on the cohesiveness, springiness and resilience of the CSBs (Figure 6D–F), which are mainly attributed to the protein quality of the dough system. In the previous report on steamed bread supplemented with certain orange fiber, the changes in springiness and cohesiveness were not completely consistent with the change in firmness [70]. Firmness seems to be more sensitive to the addition of highland barley flour than other textural parameters. The above results indicated that the addition of highland barley had no significant effect on the textural properties of the CSB.

#### 3.3.3. In Vitro Starch Digestibility

The effect of highland barley on the in vitro digestibility of wheat starch is expressed as the amount of glucose released during digestion, and Figure 6G shows the in vitro starch digestibility of the composite CSBs. The four CSB samples had similar starch hydrolysis curves, and the starch was rapidly digested in the first 30 min and then gradually digested at a slow and steady rate in the following 180 min. Importantly, the addition of three highland barley varieties decreased the starch digestibility of the CSB, and the glucose release of the JM-HLY-20 CSB decreased most significantly, while the JM-BQ-20 CSB was observed to release a high level of glucose, which was consistent with the starch digestibility characteristics reported in Section 3.2.4. This could be attributed to the reduced starch hydrolysis in the presence of higher levels of β-glucan, as β-glucan increases viscosity in the intestines, thereby slowing down starch digestibility [20,71]. In addition, phenolic compounds can be adsorbed on the starch surface to inhibit α-amylase activity, which may be another reason for the reduced starch digestibility of the composite CSBs [61]. The presence of fiber reduces the starch digestibility in the small intestine [72]. Given that there is a significant positive correlation between starch hydrolysis and glycemic index [73], the highland barley CSBs formula in this study can be served as a healthy food to slow down the increase in postprandial blood sugar.

## 4. Conclusions

The results showed that the three highland barley varieties contained different protein and starch contents, with different physiochemical and rheological properties. Therefore, the substitution of different highland barley flours at various levels for JM flour had different effects on the wheat flour and dough. Overall, highland barley increased UPP%. Although the development time and stability time of the composite doughs were reduced, the gluten micro-structure was not disrupted and became even denser. Highland barley improved the rheological properties by increasing the viscoelasticity of dough and reducing tan δ. More importantly, highland barley delayed the retrogradation of starch, which provides useful information for understanding the processing quality of the CSB formulation. The type and substitution level of highland barley had a significant effect on the quality of CSB. The JM CSB and CSB formulations with 20% highland barley substitution showed little difference in specific volume, cohesiveness, springiness and resilience. Therefore, these CSB formulations, particularly JM-HLY-20 formulation, showed good textural characteristics, similar to the JM CSB. The addition of highland barley can delay the hardening of the CSB during storage and soften their texture. Highland-barley-supplemented CSBs showed a reduced starch digestibility due to the inclusion of high TPC, TFC and β-glucan for highland barley, and the nutritional quality of the composite CSB was considerably improved. The HLY barley flour exhibited the best fortified qualities among all barley varieties, indicating great potential for the CSB supplementation. This study shows that highland barley is a promising supplement, which can improve the quality and nutritional properties of wheat products.

## Figures and Tables

**Table 1:** Parameters related to the grain quality and nutritional ingredients of wheat and three highland barley varieties.

Sample	Grain Quality Determined by Near-Infrared Reflectance			Size Distribution of B-Type Starch Granules (%)	Amylose Content (%)	H/L Ratio (%)	Nutritional Ingredient		
	Moisture Content (%)	Protein Content (%)	Starch Content (%)				β-Glucan Content (%)	Polyphenol Content (mg GAE/g)	Flavonoids Content (mg GAE/g)
JM	9.82 ± 0.08b	15.48 ± 0.19a	61.43 ± 0.56a	44.25 ± 1.38b	28.09 ± 0.32a	58.65 ± 0.60a	0.50 ± 0.06c	0.48 ± 0.09b	0.33 ± 0.08c
BQ	12.19 ± 0.12a	14.40 ± 0.33b	61.55 ± 0.79a	42.49 ± 2.50b	28.28 ± 2.25a	50.37 ± 3.01b	4.15 ± 0.10a	1.08 ± 0.09a	1.74 ± 0.09a
DLH	12.60 ± 0.09a	9.11 ± 0.19c	50.88 ± 0.75b	56.93 ± 2.64a	34.92 ± 3.60a	41.84 ± 0.61c	3.83 ± 0.11ab	1.24 ± 0.05a	1.71 ± 0.12a
HLY ^a^	5.80 ± 0.30c	15.27 ± 0.3a	47.93 ± 0.07c	47.29 ± 0.69b	32.63 ± 2.30a	24.89 ± 0.06d	3.60 ± 0.14b	1.02 ± 0.06a	1.05 ± 0.10b

Different lower-case letters in the same column show significant difference among samples (*p* < 0.05). H/L ratio: high- to low-molecular-weight glutenin ratio. ^a^ Parameters related to grain quality and nutritional ingredient of HLY have been reported previously [22].

**Table 2:** Dough-mixing properties of wheat supplemented with different amounts of highland barley flour.

Sample	Water Absorption (%)	Dough Development Time (min)	Dough Stability Time (min)	C1 (Nm)	C2 (Nm)	C3 (Nm)	C4 (Nm)	C5 (Nm)	Gelatinization Temperature (°C)
JM	64.75 ± 0.25b	6.08 ± 0.58a	8.45 ± 0.20a	1.04 ± 0.04a	0.49 ± 0.01a	1.74 ± 0.01c	1.61 ± 0.02a	2.31 ± 0.08a	78.90 ± 0.40a
JM-BQ-20	65.00 ± 0ab	4.12 ± 0.20b	7.26 ± 0.04b	1.10 ± 0.01a	0.43 ± 0c	1.69 ± 0d	0.99 ± 0c	1.37 ± 0.01c	77.05 ± 0.15b
JM-DLH-20	65.00 ± 0ab	4.73 ± 0.16b	7.53 ± 0.02ab	1.09 ± 0.01a	0.46 ± 0b	1.8 ± 0.01b	1.35 ± 0.01b	1.82 ± 0.01b	77.90 ± 0.30ab
JM-HLY-20	65.40 ± 0.20a	4.19 ± 0.02b	7.84 ± 0.04ab	1.03 ± 0a	0.47 ± 0ab	1.84 ± 0a	1.55 ± 0.01a	2.15 ± 0.03a	78.35 ± 0.15a

Different lower-case letters in the same column show significant difference among samples (*p* < 0.05). C1: dough development; C2: protein weakening; C3: starch gelatinization; C4: thermal stability; C5: final viscosity.

**Table 3:** Sensory quality of highland barley composite Chinese steamed breads.

Sample	Specific Volume	Surface Structure	Colour	Shape	Internal Structure	Elasticity	Viscidity	Flavor	Total Score
JM	18.0 ± 1.0a	8.3 ± 0.2a	9.1 ± 0.1a	8.2 ± 0.7a	13.3 ± 0.2a	14.3 ± 0.2a	9.0 ± 0a	8.9 ± 0.1a	89.0 ± 0.3a
JM-BQ-20	18.0 ± 1.0a	4.7 ± 0.2b	5.9 ± 0.1b	6.0 ± 1.0a	11.7 ± 0.2b	12.5 ± 0.5b	7.3 ± 0.3b	8.1 ± 0.1b	74.1 ± 0.5b
JM-DLH-20	16.5 ± 0.5a	5.6 ± 0.3b	6.8 ± 0.3b	6.5 ± 0.5a	12.1 ± 0b	11.2 ± 0b	7.8 ± 0.3b	8.4 ± 0.1b	74.7 ± 0.4b
JM-HLY-20	16.0 ± 1.0a	6.5 ± 0.5b	4.5 ± 0.5c	7.8 ± 0.8a	11.9 ± 0b	15.5 ± 0.5a	8.7 ± 0.2a	8.7 ± 0.2ab	79.4 ± 3.3b

Different lower-case letters in the same column show significant differences among samples (*p* < 0.05).

## Data Availability

All data generated or analyzed during this study are included in this published article.

## Supplementary Materials

The following supporting information can be downloaded at: https://www.mdpi.com/article/10.3390/foods11081091/s1, Table S1: Sensory quality scoring system for highland barley composite Chinese steamed breads.
