# Correction: Wu et al. Effect of Highland Barley on Rheological Properties, Textural Properties and Starch Digestibility of Chinese Steamed Bread. *Foods* 2022, *11*, 1091

**DOI:** 10.3390/foods14061041

**Published:** 2025-03-19

**Authors:** Daying Wu, Liwei Yu, Lei Guo, Shiquan Li, Xiaohua Yao, Youhua Yao, Xinyou Cao, Kunlun Wu, Xin Gao

**Affiliations:** 1 State Key Laboratory of Crop Stress Biology in Arid Areas and College of Agronomy, Northwest A&F University, Yangling 712100, China; wudaying@nwsuaf.edu.cn (D.W.); realylw@163.com (L.Y.); leiguo@nwsuaf.edu.cn (L.G.); lishiquan@nwsuaf.edu.cn (S.L.); 2 State Key Laboratory of Plateau Ecology & Agronomy, Qinghai Key Laboratory of Hulless Barley Genetics and Breeding, Qinghai Subcenter of National Hulless Barley Improvement, Qinghai University, Xining 810016, China; yaoxiaohua009@126.com (X.Y.); youhua8888@126.com (Y.Y.); 3 Crop Research Institute, Shandong Academy of Agricultural Sciences/National Engineering Research Center for Wheat & Maize/Key Laboratory of Wheat Biology and Genetic Improvement in North Yellow & Huai River Valley, Ministry of Agriculture/Shandong Provincial Technology Innovation Center for Wheat, Jinan 250100, China; caoxinyou@126.com

## Error in Figure

In the original publication [1], there was a mistake in Figure 2. The authors regret the mistake of misplacing the CLSM image of HLY in the place of that of BQ. The corrected version of Figure 2 appears below. The authors state that the scientific conclusions are unaffected. This correction was approved by the Academic Editor. The original publication has also been updated.

## Figures and Tables

**Figure 2:**
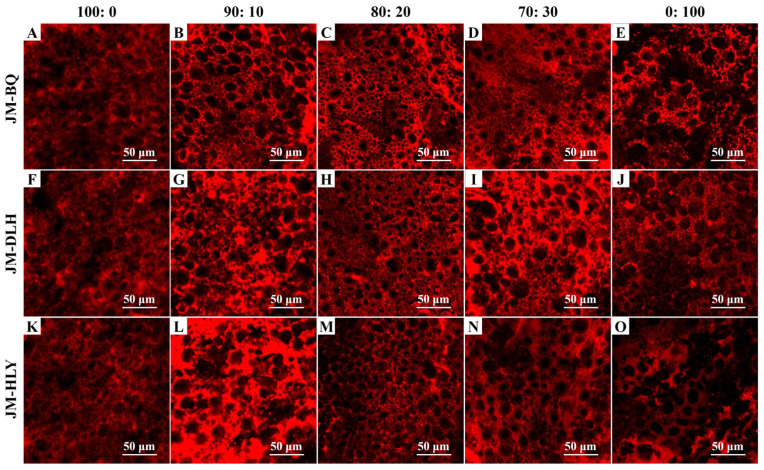
The gluten micro-structure of composite dough samples added with three highland barley varieties with different ratios: (**A**–**E**): JM-BQ; (**F**–**J**): JM-DLH; (**K**–**O**): JM-HLY. The samples stained with Rhodamine B and visualized by confocal laser scanning microscopy. Scale bar = 50 µm.
