# Supplementary material for: Effect of Highland Barley on Rheological Properties, Textural Properties and Starch Digestibility of Chinese Steamed Bread
Source: Foods. 2022 Apr 10;11(8):1091. doi: 10.3390/foods11081091 (PMC9025642; doi:10.3390/foods11081091)
Supplement: Supplementary file 1 [file foods-11-01091-s001.zip › foods-1677401-supplementary.pdf]

Table S1. Sensory quality scoring system for highland barley composite Chinese steamed breads.

Quality parameter	Score	Sensory assessment standard
Specific volume (mL/g)	20	Specific volume⩾2.8: 20;<br>2.8⩽Specific volume⩽1.5: minus 1 score per 0.1 mL/g reduction;<br>Specific volume⩽1.5: 2.
Surface structure	10	Smooth: 8.1-10;<br>Uneven, pliable, air bubble, concave or large burns: 3-8.
Colour	10	White, milky, milky white: 8.1-10;<br>Light yellow, yellow: 6.1-8;<br>Gloom, dark: 2-6.
Shape	10	Symmetrical, spherical: 7.1-10;<br>Flat, unsymmetrical: 4-7.
Internal structure	15	Longitudinal section with small pores evenly: 12.1-15;<br>Close pores, uniform or separated between the edges and the epidermis: 8.1-12;<br>Large pores, rough structure and uneven: 5-11.
Elasticity	15	Rebound quickly, can be compressed more than 1/2 after finger press: 12.1-15;<br>Rebound slowly or does not rebound after finger press: 8.1-12;<br>Difficulty pressing fingers, very hard: 3-8.
Viscidity	10	Great chewing without teeth stickness: 8.1-10;<br>Chewing is not refreshing, slightly sticky or sticky: 3-8.
Flavor	10	Wheat fragrance, no smell: 8.1-10;<br>Bland flavor: 3.1-8;<br>Unpleasant smell: 1-3.
Total	100	

Note: Scoring system: poor ≤ 70, average or general or acceptable = 70-79, good = 80-89, excellent = 90-100.
